# Human Locomotion under Reduced Gravity Conditions: Biomechanical and Neurophysiological Considerations

**DOI:** 10.1155/2014/547242

**Published:** 2014-08-28

**Authors:** Francesca Sylos-Labini, Francesco Lacquaniti, Yuri P. Ivanenko

**Affiliations:** ^1^Centre of Space Bio-Medicine, University of Rome Tor Vergata, Via Montpellier 1, 00133 Rome, Italy; ^2^Laboratory of Neuromotor Physiology, IRCCS Santa Lucia Foundation, Via Ardeatina 306, 00179 Rome, Italy; ^3^Department of Systems Medicine, University of Rome Tor Vergata, Via Montpellier 1, 00133 Rome, Italy

## Abstract

Reduced gravity offers unique opportunities to study motor behavior. This paper aims at providing a review on current issues of the known tools and techniques used for hypogravity simulation and their effects on human locomotion. Walking and running rely on the limb oscillatory mechanics, and one way to change its dynamic properties is to modify the level of gravity. Gravity has a strong effect on the optimal rate of limb oscillations, optimal walking speed, and muscle activity patterns, and gait transitions occur smoothly and at slower speeds at lower gravity levels. Altered center of mass movements and interplay between stance and swing leg dynamics may challenge new forms of locomotion in a heterogravity environment. Furthermore, observations in the lack of gravity effects help to reveal the intrinsic properties of locomotor pattern generators and make evident facilitation of nonvoluntary limb stepping. In view of that, space neurosciences research has participated in the development of new technologies that can be used as an effective tool for gait rehabilitation.

## 1. Introduction

Life evolved in the presence of gravity, which has two major impacts on motor functions: specific body orientation in space and antigravity muscle tone and specific rules of motion in the gravity field. Gravity plays an essential role in terrestrial locomotion. The dominant hypothesis regarding templates for bipedal walking in the gravity field is the pendular mechanism of walking, up to intermediate speeds, and the bouncing mechanism of running, up to the highest speeds attainable [1]. The inverted pendulum-like mechanism of energy exchange taking place during walking would be optimized at slower speeds in reduced gravity [2, 3]. Despite our intuitive appreciation for the influence of gravity, we do not fully understand how gravity interacts with other forces, such as inertia, to affect many biological and physical processes and what type of gait and/or limb synchronization (trot, gallop, lateral sequence walk, pace, skipping, etc.) would evolve at other gravity levels.

Understanding locomotion characteristics is critical for those working in the area of gait biomechanics and neurophysiology of pattern generation networks and of exercise countermeasures for astronauts. Many researchers have investigated the effects of reducing and eliminating gravity on locomotive kinematics and kinetics [4–8]. Others have studied locomotion in actual weightlessness or hypogravity [9, 10]. The techniques have included supine and erect cable suspension, parabolic aircraft flights, water immersion, and centrifugal methods [6]. Increased knowledge of locomotion kinematics, kinetics, muscular activity patterns, and sensory feedback modulation may help to facilitate more effective exercise countermeasures, develop innovative technologies for gait rehabilitation, and provide new insights into our understanding of the physiological effects of gravity. In this review, we will consider the known tools and techniques used for hypogravity simulation and their effects on human locomotion.

## 2. Methods and Apparatuses for Reduced Gravity Simulation

Spaceflights are the more direct way to assess the effect of gravity on locomotion, but studying locomotion in actual hypogravity is demanding and expensive [6]. The drawbacks to spaceflight experiments include difficulty in using necessary data collection hardware and performing an experiment with adequate sample size. Parabolic flight offers a viable alternative, but periods of weightlessness are limited to ~20 s, which only allows for acute locomotion investigations [11].

**Figure 1:**
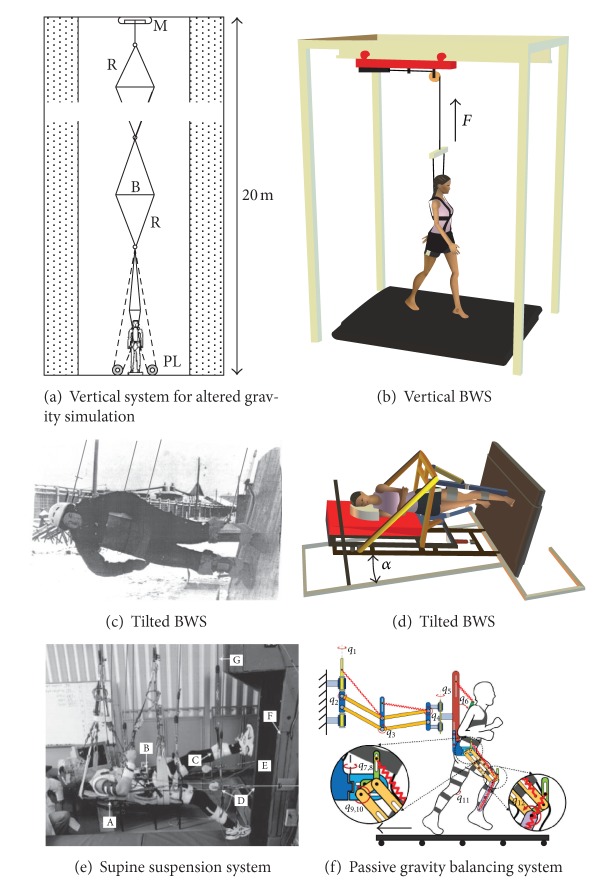
Reduced gravity simulators for locomotion. (a) Schema of the vertical system used to simulate different gravity values (redrawn from [8]). R: rubber bands, B: light metal bars, M: electric motor to stretch the elastic band system, PL: pulleys to invert the direction of the pull on the subject (dashed lines). (b) Vertical body weight support (BWS) system: subject walks on a treadmill with different levels of BWS while being supported in a harness, pulled upwards by a preset unloading force *F*. (c) Tilted BWS system used by Roscosmos (Russian Federal Space Agency) to train astronauts before space flights [15]: the subject walks on a truncated cone (60 m height, 9.2° inclination relative to the vertical), supported by five ropes sustaining the head, trunk, and legs (picture portraying Professor Gurfinkel reproduced with his kind permission). (d) Tilted unloading system for stepping on a treadmill: the subject lies on the side on a tilted couch (up to 40° from the horizontal position) with both legs suspended in the exoskeleton and steps on the treadmill, which is tilted to the same angle. The component of the gravity force acting on the stance and swing limb segments is proportional to the tilting angle α [18]. (e) Supine suspension system (adapted from [20], courtesy of Professor Peter Cavanagh): the subject is suspended horizontally attached to latex rubber cords. A cloth sleeve and rubber cord are attached each to the upper and lower arms and legs (eight total). The subject is actively pulled toward the treadmill by a gravity replacement load through cables attached to a load splitter. (f) Passive reduced gravity walking simulator (courtesy of Dr. Ou Ma). The system consists of a 3-DOF dual parallelogram mechanism, a 2-DOF torso support assembly, and a pair of 3-DOF leg exoskeletons. The weight of the body and the legs is compensated by the spring-balanced dual-parallelogram mechanism and torso-support assembly, and the weight of each leg is compensated by a leg exoskeleton [22–24].

There are several apparatuses that have been used in the past to simulate reduced gravity locomotion. One of the more used systems is the vertical body weight support (BWS) (Figures 1(a) and 1(b)). These kinds of simulators are usually obtained supporting the subjects in a harness that applies a controlled upward force. For example, the WARD [12] mechanism consists of a mechanical gear driven by a pneumatic cylinder (Figure 1(b)). It is held in a cart that slides forward and backward over a track. Low-friction sliding of the mechanism ensures that only vertical forces are applied to the subject. Vertical BWS systems may also make use of a small increase in air pressure around the user's lower body to create a lifting force approximately at the person's center of mass [13]. Other vertical systems [8, 14] use a series of compliant rubber spring elements that are stretched to create the upward (to simulate gravity less than 1 g) or downward (to simulate gravity greater than 1 g) force (Figure 1(a)). The main limitation of these reduced gravity simulators (in addition to high local skin pressure via a harness) is that each supporting limb experiences a simulated reduction of gravity proportional to the applied force, while the swinging limb experiences 1 g.

The tilted BWS systems (Figures 1(c) and 1(d)) are constructed to simulate more realistic effects of gravity changes on both the stance and swing legs in the sagittal plane. These simulators, that have been used in the past by both Roscosmos (Russian Federal Space Agency) and NASA to train astronauts before space flights [15–17], are based on the idea of neutralizing the component of the gravity force normal to the lying surface [mg · cos(α), where α is the angle of inclination], while the component of the gravity force acting on the body and swinging limbs in the sagittal plane is reduced in relation to the tilt angle [mg · sin(α)]. A similar concept has been used in the reduced gravity simulator (Figure 1(d)) designed by Ivanenko et al. (Italian patent number Rm2007A000489): the subject lies on the side on a tilted couch (up to 40° from the horizontal position) with both legs suspended in the exoskeleton and steps on the treadmill, which is tilted to the same angle [7, 18, 19]. This simulator included additional mass of the tilted chassis (~15 kg) and exoskeleton (1.5 kg for each leg). Thus the entire assembly had a mass of ~18 kg that increased both gravitational and inertial forces during walking.

Another class of gravity-related manipulations is “subject load device” (SLD) that applies a gravity replacement force in the direction down to the surface. This type of SLD can be used in the vertical systems to increase the gravity [8] or in the lying position (Figure 1(e)). When an astronaut walks or runs on a treadmill in weightlessness, a subject load device is used to return him or her back to the treadmill belt and to load the limbs. The gravity replacement load is transferred, via a harness, to the pelvis and/or the shoulders. Gravity simulators can simulate active treadmill running in weightlessness and provide a method of testing proposed improvements in SLD design and exercise protocols [20, 21]. In supine suspension systems (Figure 1(e)), subjects are suspended horizontally attached to latex rubber cords. A cloth sleeve and rubber cord are attached each to the upper and lower arms and legs (eight total) [20]. The limitation of this system is a local pressure on some parts of the body (e.g., shoulders) and modifications in the swing phase dynamics due to nonconstant forces of rubber cords and gravity acting in the anterioposterior direction of leg movements (Figure 1(e)).

Based on the passive gravity balancing technology, Ma et al. [22, 23] proposed a design concept of a passive reduced gravity simulator to simulate human walking or other activities in a reduced-gravity environment for potential applications of training astronauts and space travelers (Figure 1(f)). The system consists of a 3-DOF dual parallelogram mechanism, a 2-DOF torso support assembly, and a pair of 3-DOF leg exoskeletons. The weight of the body and the legs is compensated by the spring-balanced dual-parallelogram mechanism and torso-support assembly, and the weight of each leg is compensated by a leg exoskeleton. The system is capable of simulating human walking and jumping in a hypogravity environment [24]. Hardware prototyping and experimental study of the new system are currently underway.

In the following section we discuss the basic principles of adaptation of locomotion to different gravity values using the technologies described here.

## 3. Biomechanical Aspects of Locomotion in Reduced Gravity 

**Figure 2:**
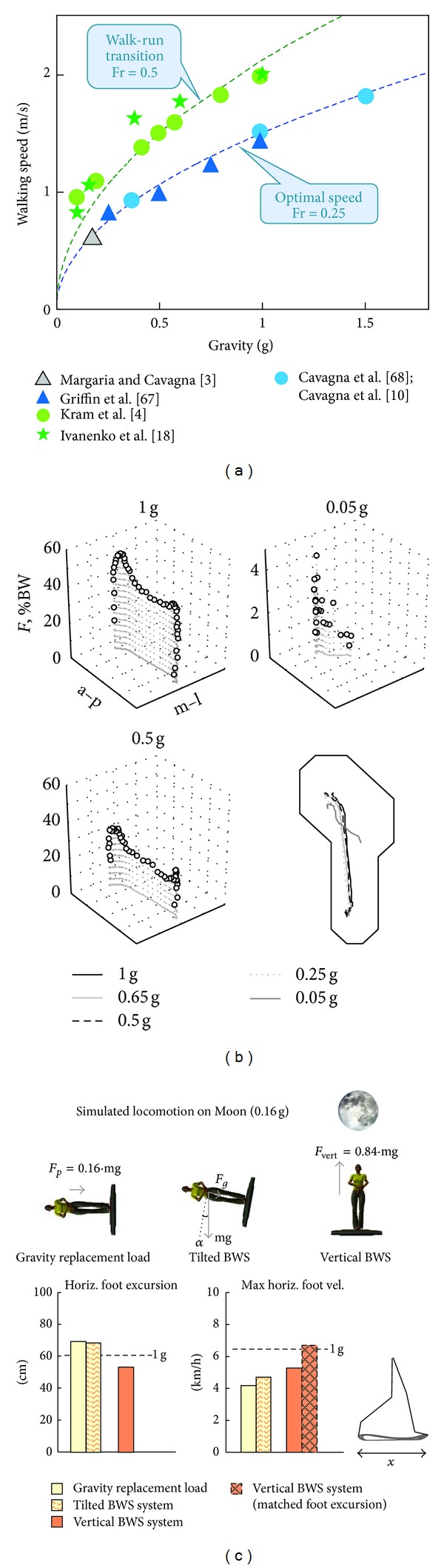
Biomechanical features of locomotion in reduced gravity conditions. (a) Optimal (blue) and walk-to-run transition (green) speeds as a function of gravity. Dynamically similar speeds predicted by Fr = 0.25 and Fr = 0.5 are indicated by blue and green dashed curves, respectively [25]. Green circles and stars refer to measurements of optimal walk-to-run transition speeds in simulated low-gravity conditions [5, 18]. The grey triangle indicates an earlier estimate of optimal walking speed predicted for the Moon gravitational environment by Margaria and Cavagna [3]. Blue triangles refer to the optimal speeds (at which most of the mechanical exchange between potential and kinetic energy of the body center of mass occurs) obtained in a simulation study of Griffin et al. [26]. Blue circles represent measurements of optimal speed obtained during parabolic flight [10, 27]. (b) Time course of the net vertical component of in-shoe reaction forces plotted as a function of the spatial coordinates of the foot at different reduced gravity levels. Note change in vertical scale in the 0.05 g condition. The lower right panel shows the trajectories of the center of pressure superimposed on a foot outline (adapted from [28]). (c) Maximum longitudinal foot velocity and foot excursion (*x*) during walking at 2 km/h at 0.16 g using three different reduced gravity simulators (represented schematically in the upper panels). Horizontal dashed lines indicate values for walking at 1 g. The hatched bar (right panel) corresponds to the maximal foot velocity for the vertical BWS system approximated by matching the foot excursion to that of the tilted BWS system [7]. Note significantly lower foot velocities during swing using tilted BWS systems.

Despite some differences, all reduced gravity simulation approaches show a reasonable approximation of the reduction in the gravitational force acting on the center of body mass (COM) and similar results concerning the speed of gait transitions. An important consequence of the pendulum-like behavior of the limbs in the gravity field is the principle of dynamic similarity [29], which states that geometrically similar bodies that rely on pendulum-like mechanics of movement have similar gait dynamics at the same Froude number:

(1) $$\begin{array}{c} \text{Fr}\;\sim \frac{V^{2}}{gL}, \end{array}$$

where *V* is the speed of locomotion, *g* is the acceleration of gravity, and *L* is a characteristic leg length. That is, all lengths, times, and forces scale by the same factors. In order to optimize the recovery of mechanical energy, the kinetic energy and the potential energy curves must be equal in amplitude and opposite in phase, as in a pendulum. Assuming that the change in kinetic energy within each step is an increasing function of the walking speed (while the change in the potential energy is proportional to gravity), the hypothesis was proposed that the inverted pendulum-like mechanism of energy exchange during walking would be optimized at slower speeds in reduced gravity [3, 10]. An optimal exchange between potential and kinetic energies of the COM occurs at Fr ~ 0.25 [2] (Figure 2(a)). Even though specific limb segment proportions may play an essential role in the kinematics and energetics of walking [30], animal anatomy and individualized limb segment dimensions are optimized in such a way that the Froude number can explain optimal walking velocity.

On Earth, walking and running gaits are usually adopted for different speeds of locomotion, with a preferred transition occurring at ~2 m/s for human adults and at slow speeds for children (Fr ~ 0.5), in accordance with the dynamic similarity theory [29]. Different studies [4, 18] demonstrated that, at lower levels of gravity, the walk-run transition occurred at progressively slower absolute speeds but at approximately the same Froude number (Figure 2(a)).

Despite similarities in approximating reduced gravity, there are nevertheless essential differences between different simulation approaches. The variables that showed the greatest differences between vertical and tilted reduced gravity systems (Figure 1) were maximal longitudinal foot velocity and longitudinal foot excursion (Figure 2(c)), in agreement with significant influences of gravity on swing leg dynamics [7]. Even though the maximal longitudinal foot velocity for the tilted BWS condition decreased only slightly relative to the vertical BWS, however, the actual decrement was much more obvious if one takes into account that it was significantly compensated for or masked by increments in the stride length [7]. A previous modeling study also predicted differential effects of gravity during stance and swing phases [31]. In fact, the changes in the longitudinal foot excursion were basically opposite for the vertical and tilted BWS systems (Figure 2(c)). For the former system the amplitude of longitudinal foot motion decreased, while for the latter system it increased relative to the 1 g condition. Considering a monotonic (presumably proportional [32]) relationship between the stride length and the maximal foot velocity at a given gravity level (1 g), the peak foot velocity would be expected to be ~1.5 times higher for the vertical than for tilted BWS condition if the stride lengths were similar (Figure 2(c)). The previous studies on parabolic flights investigating the effect of gravity on walking mechanics demonstrated increments in the swing phase duration (by 29% at 0.25 g [33]; see also [11]), in line with the substantial contribution of gravity to the swing leg. Overall, the findings demonstrate that gravity acting on both stance and swing legs plays an important role in shaping locomotor patterns.

## 4. Nonlinear Reorganization of EMG Patterns

It is known that load plays a crucial role in shaping patterned motor output during stepping [34–36], and humans produce a specific heel-to-toe rolling pattern during stance in normal gravity conditions. Ground contact forces reflect the net vertical and shear forces acting on the contact surface and result from the sum of the mass-acceleration products of all body segments while the foot is in contact with ground [37]. Simulating reduced gravity between 0.05 and 1 g reveals drastic changes of kinetic parameters but limited changes of the kinematic coordination [28]. The reported accurate control of limb/foot kinematics [28] may depend on load- and displacement-compensation mechanisms working effectively throughout a wide range of ground contact forces, from full body weight up to <5% of its value. The peak vertical contact forces decrease proportionally to gravity, but at 0.05 g they are applied at the forefoot only (Figure 2(b)). During lower limb loading, a variety of receptors can be activated, such as Golgi tendon organs, cutaneous receptors of the foot, and spindles from stretched muscles [36]. These sensory signals interact with central rhythm-generating centers and help in shaping the motor patterns, controlling phase-transitions, and reinforcing ongoing activity [38, 39]. For instance, loading of the limb enhances the activity in antigravity muscles during stance and delays the onset of the next flexion [40]. It is important to understand the mechanisms of sensorimotor adaptation to the biomechanics of locomotion and foot placement/loading in heterogravity, especially to longer-term changes of load.

**Figure 3:**
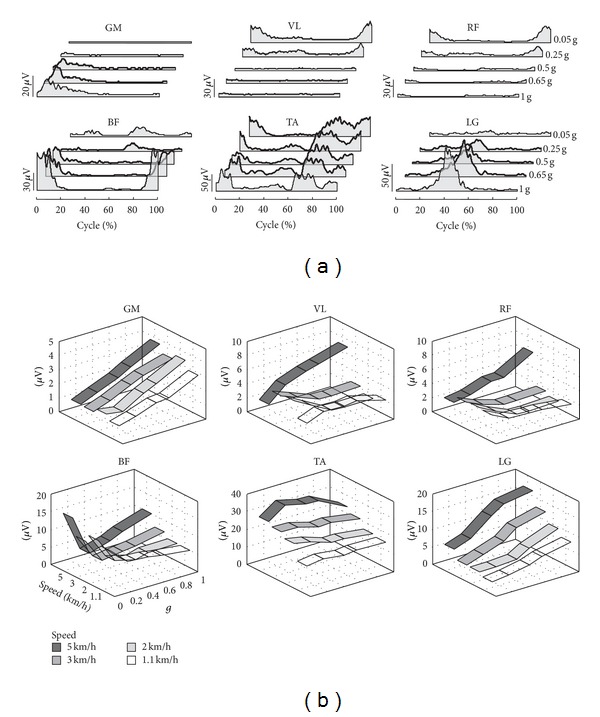
Nonlinear reorganization of muscle activity patterns. (a) An example of ensemble-averaged electromyographic (EMG) activity of lower limb muscles versus the normalized gait cycle is shown for a subject walking at 3 km/h at different simulated reduced gravity levels [28]. (b) Mean EMG activity computed over the gait cycle and averaged across all cycles and subjects (*n* = 8). For each muscle, values for trials performed at each speed are plotted as a function of simulated reduced gravity (adapted from [28]). GM, gluteus maximus, VL, vastus lateralis, RF, rectus femoris, BF, biceps femoris, TA, tibialis anterior, LG, and lateral gastrocnemius.

A key feature of adaptation to hypogravity is a remarkable nonlinear scaling of muscle activity patterns contrary to monotonic changes in foot loading. The simplest kind of change with simulated reduced gravity [28] was seen in ankle extensors: the mean amplitude of activity decreased systematically with decreasing simulated gravity, consistent with their antigravity function [35, 41]. By contrast, the behavior of other muscles could not be predicted simply on the basis of the static load during stance. The amplitude and pattern of muscle activity generally depended on speed and could vary nonmonotonically with body unloading. There was also a complex reorganization of the pattern of activity of thigh muscles with decreasing simulated gravity, as well as noteworthy individual differences [28].  Figure 3(a) illustrates an example of nonlinear reorganization of EMG patterns in one subject walking at 3 km/h. With body weight unloading, gluteus maximus and distal leg extensors decreased their activity, while other muscles demonstrated a “paradoxical” increment of activation (e.g., quadriceps) or considerable changes in the activation waveforms (hamstring muscles). Note also the absence of the typical burst of RF at the beginning of the swing phase at low simulated gravity levels (Figure 3(a)), consistent with other studies on the effect of body weight unloading [42] and walking speed [43]. It is unlikely that these changes are due to the order of trials or the consequence of learning the hypogravity condition since presentation order of speeds and BWS was randomized across sessions and experiments [28]. Also, the duration of each trial was ~1 min, with at least 2 min rest between trials, and a short (~30 s) training period of walking at different speeds was allowed for each simulated reduced gravity level before the actual data collection was begun (the walking patterns typically adapt rapidly to simulated reduced gravity [4, 5]). This reorganization is presumably related to the multifunctional (biarticular) action of these muscles and to the need to repartition the joint torque contributions across different muscles as a function of the changes induced by gravity. At 1 g, the main peak of m. biceps femoris activity occurring before heel-contact serves to decelerate the swinging limb [37]. However, as gravity is decreased, its main activity occurs in mid-stance and late stance, presumably in relation to the need to assist vaulting over an inverted pendulum of the stance limb and swing initiation.

There might be various factors accounting for the nonlinear reorganization of muscle activity patterns with gravity. To start with, nonlinear scaling also occurs during walking at different speeds at 1 g. For instance, VL and RF activity is quite small at low speeds (less than ~3 km/h) but becomes prominent at higher speeds (>4 km/h) (Figure 3(b)), a speed effect consistent with that reported in the literature [28, 43, 45, 46]. Given that, it should be stressed that walking at lower gravity levels at the same speed (Figure 3(a)) corresponds to walking at higher speeds if one uses the Froude number as a dimensionless parameter (e.g., walk-run transition at 0.25 g occurs at ~4 km/h, Figure 2(a)), so that “paradoxical” increments of VL and RF EMG activity in Figure 3(a) may reflect higher biomechanical demands on proximal leg muscles at higher dimensionless speeds. Nonlinear reorganization of EMG patterns was also observed when using exoskeleton robotic devices that provide body weight support [42, 47]. Changes in the body reference configuration during stance (slightly flexed posture [48, 49]) may contribute to a greater activity of proximal extensors as well. Finally, there is a differential effect of speed on quadriceps muscle activity at reduced gravity levels: VL and RF activity increases at low speeds (<3 km/h) while it decreases at a high speed (5 km/h) (Figure 3(b)). Potential nonlinear scaling of muscle activity for most whole body movements in microgravity should also be taken into account for exercise countermeasures for astronauts.

## 5. Different Gaits

Considering complex, high-dimensional, dynamically coupled interactions between an organism and gravitational environment, in principle, one challenging solution is to adopt different coordination patterns and not only an optimal speed of locomotion. Are different gaits possible on other planets?

**Figure 4:**
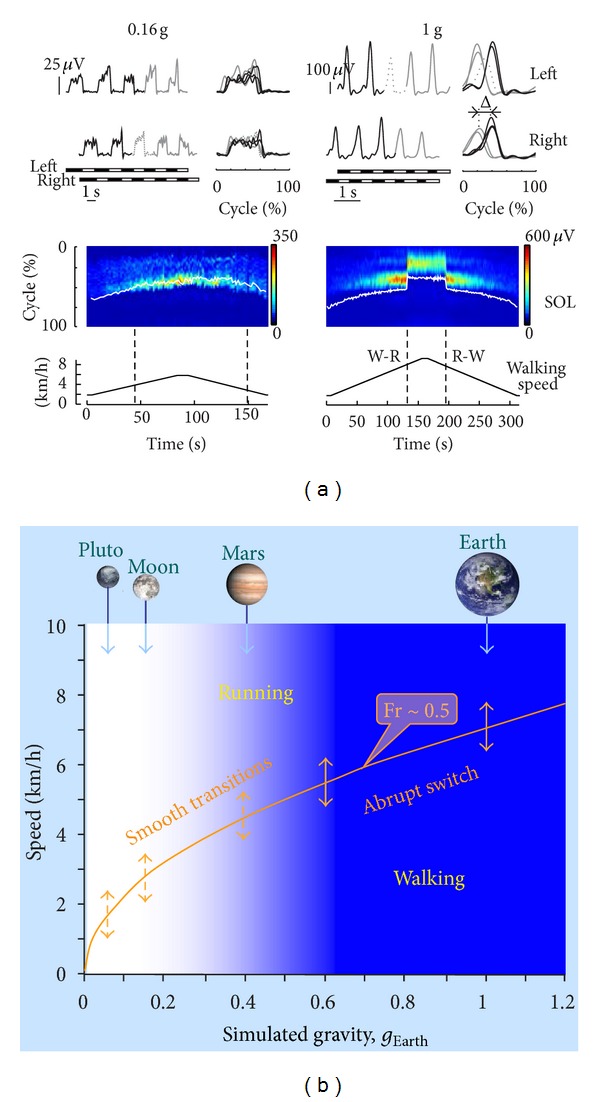
Smoothness/abruptness of gait transitions at different gravity levels. (a) Soleus (SOL) EMG patterns during slow changes in treadmill belt speed (lower panels) in one representative subject at 0.16 g (left) and 1 g (right).* Upper panels*: examples of SOL EMG waveforms (left, plotted versus time; right, plotted versus normalized cycle) during 5 consecutive strides of both legs around the transition from walking (black lines) to running (gray lines). Dotted curves denote the (transition) stride of the leg in which the swing phase first exceeded 50% gait cycle. Bottom horizontal bars denote stance (black) and swing (white) phases.* Lower panels*: the color maps represent a sequence of discrete activation waveforms (vertical slices). *x*-axis indicates the number of the gait cycles (corresponding to the appropriate timing of the trial), *y*-axis indicates normalized gait cycle (from touchdown to another touchdown), and color indicates EMG amplitude. The white line indicates when toe off occurred. Vertical dashed lines indicate walk-to-run (W-R) and run-to-walk (R-W) transitions. Note abrupt changes in the relative stance duration and muscle activation patterns at gait transitions at 1 g and no obvious distinction in these parameters at the transition from walking to running at 0.16 g. (b) Schematic representation of the smoothness of gait transitions as a function of gravity. The orange curve symbolizes the dimensionless walk-run transition speed consistent with the theory of dynamic similarity (Fr ~ 0.5) [19, 29, 44]. The blue color range of gravitational levels represents a discontinuous switch from walk to run, whereas the white region indicates smooth transitions.

One approach to study locomotor adaptations is to look at the effect of gravity on gait transitions. A gait has been defined as “a pattern of locomotion characteristic of a limited range of speeds described by quantities of which one or more change discontinuously at transitions to other gaits” [29]. An important aspect of gait transitions is a discontinuous switch that occurs at some point while varying the speed of progression (although some exceptions may exist [50–52]). As already discussed (Figure 2(a)), gravity has a strong effect on the speed at which gait transitions occur (Fr ~0.5). Surprisingly, however, we found [18, 19] that at lower levels of simulated gravity the transition between walking and running was generally gradual, without any noticeable abrupt change in gait parameters or EMG bursts (Figure 4(a)). This was associated with a significant prolongation of the swing phase, whose duration became virtually equal to that of stance in the vicinity of the walk-run transition speed, and with a gradual shift from inverted-pendulum gait (walking) to bouncing gait (running). A lack of discontinuous changes in the pattern of speed-dependent locomotor characteristics in a hypogravity environment (Figure 4(b)) is consistent with the idea of a continuous shift in the state of a given set of central pattern generators, rather than the activation of a separate set of central pattern generators for each distinct gait [19].

Interestingly, the smoothness of gait transitions is accompanied by a gradual shift from inverted-pendulum gait to bouncing gait, resulting in a “paradoxical” inverted-pendulum running in the vicinity of run-walk and walk-run transitions [18]. The swing phase may have more influence on gait than it was previously thought. For instance, relatively slower swing and longer foot excursions (tilted BWS condition, Figure 2(c)) may raise questions about optimality or comfort of walking and could account for potentially different preferred gaits, such as loping on the Moon observed in Apollo astronauts (though the Lunar suit limits the range of motion in the leg joints and may also contribute to the loping gait on the Moon [9]). The resulting changes in the intersegmental and interlimb coordination may in turn affect the COM motion. Overall, the results support the idea of looking for new forms of locomotion (both bipedal and quadrupedal) in a heterogravity environment [54] based on the interplay between stance and swing leg dynamics, altered interlimb coupling, and altered center of mass movements.

Other significant influences of gravity on short-term and long-term gait adaptations may be related to its effects on the body reference configuration [48, 49] and anticipatory mechanisms of limb and body movements [55, 56]. For instance, the basis of habitual human posture is postural tone of the skeletal muscles and microgravity elicits substantial changes in muscle tone and posture [48, 49]. Based on clinical observations, it has been recently argued that any reflection on the nature and choice of preferred gait (e.g., bipedal versus quadrupedal) should include a consideration of the mechanisms determining the choice of unconscious habitual posture [57]. Also, in analogy with the results based on upper-limb movements related to time-to-contact [55] or movement planning [58], anticipatory postural and locomotor adjustments for lower limb movements (e.g., for the control of heel strike or accurate foot placement) should take gravity into consideration. Therefore, altered gravity conditions may also affect locomotor-related tasks such as the negotiation of stationary and moving obstructions during walking or gait initiation/termination [56, 59, 60].

**Figure 5:**
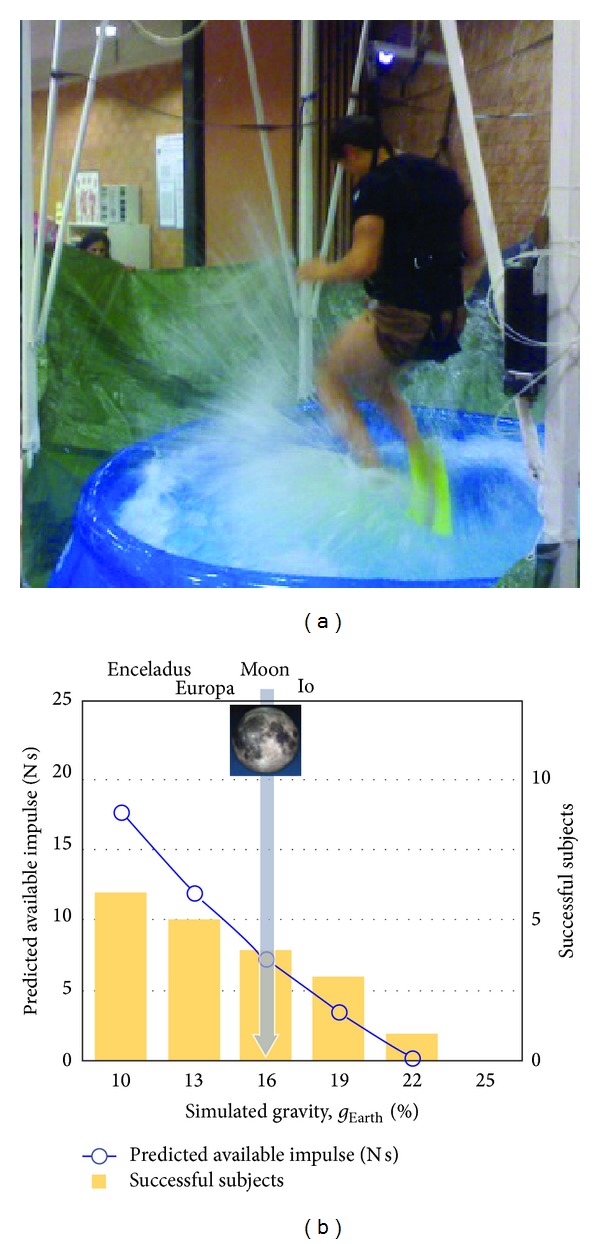
Running on water at simulated reduced gravity. The blue curve represents the net vertical impulse available to run on water, as predicted by the model used by Minetti et al. [53]. Bars represent the number of subjects, out of 6, capable of avoiding sinking at different simulated gravity values. Both variables show that 22% of Earth gravity (*g*_EARTH_) is the maximum gravity at which humans can run on water, when assisted by a small rigid fin (as illustrated in the left panel).

Finally, the repertoire of known gaits can be expanded to a variety of animals. For instance, on Earth only a few legged species, such as water strider insects and some aquatic birds and lizards, can run on water. For most other species, including humans, this is precluded by body size and proportions, lack of appropriate appendages, and limited muscle power. However, if gravity is reduced to less than Earth's gravity, running on water should require less muscle power. Recently, Minetti et al. [53] used this hydrodynamic model of Glasheen and McMahon [61] to predict the gravity levels at which humans should be able to run on water and tested the hypothesis in the laboratory using a reduced gravity simulator (Figure 5). The results showed that a hydrodynamic model of Basilisk lizards running on water [61] can also be applied to humans, despite the enormous difference in body size and morphology. Particularly, 22% of Earth's gravity is the maximum at which humans can run on water, when assisted by a small rigid fin (Figure 5) [53]. It is also worth noting the limitations for our musculoskeletal system for producing force/power (endurance); for instance, the stride frequency in humans is limited to about 2 Hz, whatever the planet is. On Earth, the biggest animal that can run on water is likely Western Grebes, and even these birds can run only for several seconds since the force production is basically anaerobic (participants in [53] could run at simulated “Moon” gravity only for ~10 s). In contrast, at reduced gravity (Moon), these birds could run on water in a charming manner for much longer time.

## 6. Clinical Implications 

Reduced gravity also offers unique opportunities for adjusting the basic patterns to altered locomotor conditions for gait rehabilitation. Body weight support systems coupled with robotic devices or pharmacologic treatments are now often used in the rehabilitation practice to assist physical therapy of individuals with neurological disorders. We will not review any detailed analysis of clinical outcomes for ambulation when using locomotor training with body weight support systems and refer to other reviews [64]. Nevertheless, it is worth emphasizing a facilitatory effect of the lack of gravity on rhythmogenesis and its potential for gait recovery.

**Figure 6:**
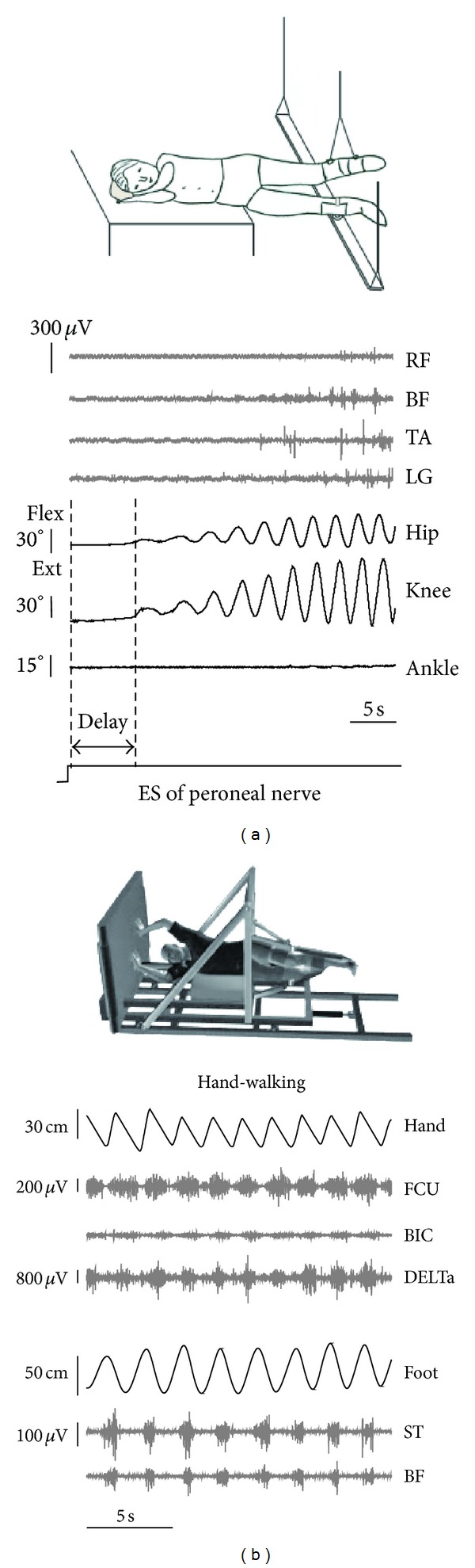
Eliciting nonvoluntary limb stepping movements in simulated weightlessness (gravity neutral) conditions. (a) An example of nonvoluntary rhythmic movements of the suspended legs induced by electrical stimulation (ES) of peroneal nerve from the study of Selionov et al. [62]. Note the absence of ankle joint rotations during evoked air-stepping. (b) An example of evoked rhythmic leg movements during hand walking in one subject from the study of Sylos-Labini et al. [63]. RF, rectus femoris, BF, biceps femoris, TA, tibialis anterior, LG, lateral gastrocnemius, FCU, flexor carpi ulnaris, BIC, biceps brachii, DELTa, anterior deltoid, ST, and semitendinosus. Hand and foot denote anterior-posterior displacements of the left hand and foot.

Novel pharmacological strategies [65] and electromagnetic stimulation techniques [62, 66–68] are being developed aimed at modulating spinal activity and restoring the locomotor function. The spinal central pattern generator (CPG) circuitry can be easily activated in healthy humans in a gravity neutral position by applying tonic central and peripheral sensory inputs. To minimize interference with the ongoing task of body weight and balance control, stepping movements are elicited during air-stepping in the absence of gravity influences and external resistance.  Figure 6 illustrates examples of nonvoluntary rhythmic movements of the suspended legs induced by electrical stimulation of peroneal nerve [62] and during hand walking [63]. It has been suggested that functional multisensory stimulations and a functional neural coupling between arm and legs can inspect CPG access by sensory and central activations and entrain locomotor neural networks and promote gait recovery. Such investigations may contribute to the clinical development of central pattern generator-modulating therapies and neuroprosthetic technologies [65, 69].

## 7. Concluding Remarks

This perspective outlines an interdisciplinary approach to extend our knowledge on adaptation of human locomotion to a hypogravity environment, including biomechanical, neurophysiological, and comparative aspects, effective exercise countermeasures for astronauts, and even exobiology of new forms of locomotion on different planets. The tools and techniques used for hypogravity simulation and their effects on human locomotion provide new insights into our understanding of the physiological effects of gravity. The beneficial effect of weightlessness on rhythmogenesis would further enhance the utility of this approach and developments of innovative technologies for gait rehabilitation.
